# Importance of Skeletal Staging in Chondrosarcoma of Bone: Results of Survey on Current Practices Among Musculoskeletal Oncologists

**DOI:** 10.1007/s43465-020-00125-3

**Published:** 2020-05-06

**Authors:** Ashish Gulia, Srinath Gupta, Vineet Kurisunkal, Ajay Puri

**Affiliations:** Department of Surgical Oncology, Tata Memorial Hospital, HBNI, Mumbai, 400012 India

**Keywords:** Chondrosarcoma, Staging, Bone scan, FDG-PET–CT, Survey analysis

## Abstract

**Purpose:**

There are no clear guidelines for staging of conventional chondrosarcoma. We conducted an online survey to determine the current practices for skeletal staging for conventional chondrosarcoma among practicing oncologists and to assess any discrepancy in practices and with the published literature.

**Methodology:**

A simple ten-question online survey (e-mails and WhatsApp) was conducted among practicing oncologists over a period of 3 weeks using online portal (surveymonkey.com). It was followed by analysis based on each question to find current practices.

**Results:**

139 members participated in the survey (84% surgeons, 9% radiologists, 3% medical and 3% radiation oncologists and 1% nuclear medicine). 65% have been treating chondrosarcoma for more than 5 years. 88% opined that biopsy is mandatory even if the radiology is suggestive of a chondrosarcoma. 66% said that solitary skeletal metastasis is seen in less than 2% of the cases but 84% of participants were in favour of performing an investigation (bone scan/PET scan) for skeletal survey. While 43% opined skeletal metastasis is more common in recurrent chondrosarcoma, 26% said that performing a bone scan was likely to impact management, 28% said it will not impact management and 46% were unsure. Of the group who thought that a bone scan would impact management or were unsure, the majority (56%) opined that this was relevant only in grade 2 and grade 3 chondrosarcoma.

**Conclusion:**

There was lack of consensus regarding staging for chondrosarcoma. Only 26% of respondents were convinced that performing a bone scan was likely to impact management of chondrosarcoma. There is a need to analyze large data sets (retrospective/prospective) to arrive at an evidence-based staging algorithm for chondrosarcoma.

## Introduction

Chondrosarcomas are the second most common solid malignant tumors of bone, commonly seen after the age of 40 years [1, 2]. They are a heterogeneous group which share in common the production of chondroid matrix [3]. Chondrosarcomas can vary from being low-grade, slow-growing lesions with low metastatic potential to high-grade aggressive lesions. Staging plays an integral part in management of bone sarcomas as the intent of treatment depends on results of staging. In cases with widespread metastatic lesions, the intent of treatment would be palliative and limb salvage may not be attempted as the prognosis is poor. Current NCCN or ESMO guidelines have suggested using bone scan with a non-contrast computerized tomography of the thorax (NCCT) or a fluorodeoxyglucose positron emission tomography (FDG-PET) scan for staging in chondrosarcoma similar to other sarcomas [4, 5]. There is limited literature available on staging in chondrosarcoma with a few reports mentioning the incidence of bone metastasis to be less than 1% [6].

Considering the low incidence of bony metastasis, we conducted a survey to assess the opinion of practicing musculoskeletal oncologists as regards the prevalent beliefs about metastasis and staging practices in chondrosarcoma. The present study describes the results of this survey and suggests future avenues for research.

## Materials and Methods

We conducted an online survey using a web-based platform “Survey Monkey” which helps in conducting customizable surveys including data analysis. The survey comprised of ten questions ranging from presentation to workup of chondrosarcoma (conventional chondrosarcomas—Grades 1, 2 and 3). The survey was conducted over a duration of 3 weeks and was circulated to medical professionals involved in the evaluation and management of chondrosarcoma. These included professionals from various subspecialties—orthopedic oncology, medical oncology, radiation oncology, musculoskeletal radiology, pathology and nuclear imaging.

The information was circulated by e-mail and WhatsApp to practicing sarcoma specialists from various international sarcoma centers and members of the Indian Musculoskeletal Oncology Society. The introductory note explained the purpose of our survey and a link to the web-based questionnaire. The questionnaire contained ten pages comprising of ten mandatory questions. These questions were formulated by the senior authors with an aim to obtaining relevant objective information regarding the experience and principles of management of chondrosarcoma across different subspecialties.

The questions asked were as follows:

1. Which specialty do you belong to?
2. How many years after specialty qualification have you been in practice?
3. How many cases of chondrosarcoma do you see in a year?
4. If the radiological findings suggest chondrosarcoma, is a biopsy mandatory before final surgery?
5. How do you stage skeletal chondrosarcoma?
6. What is the most common site for distant metastasis in skeletal chondrosarcoma you have encountered?
7. How often do you see bony metastasis only (in the absence of pulmonary metastasis) in chondrosarcoma?
8. Is bony metastasis more frequently associated with recurrent/intervened or cases associated with pathological fracture?
9. Will omitting a bone scan from staging investigations of chondrosarcoma impact management?
10. If yes/may be, do you feel bone scan as a staging modality is mandated only for Grade II or Grade III chondrosarcoma?

Each question had four options, out of which the participants had to choose one option. Two reminders were sent after 7 and 14 days. The survey closed at 3 weeks.

Of the four options given for each questions, the one with maximum votes has been considered for discussion as it highlights the general consensus of the participants. Those results which were unconventional were also made a note of and compared with the literature. Incomplete surveys were excluded from the final analysis.

## Results

**Fig. 1:**
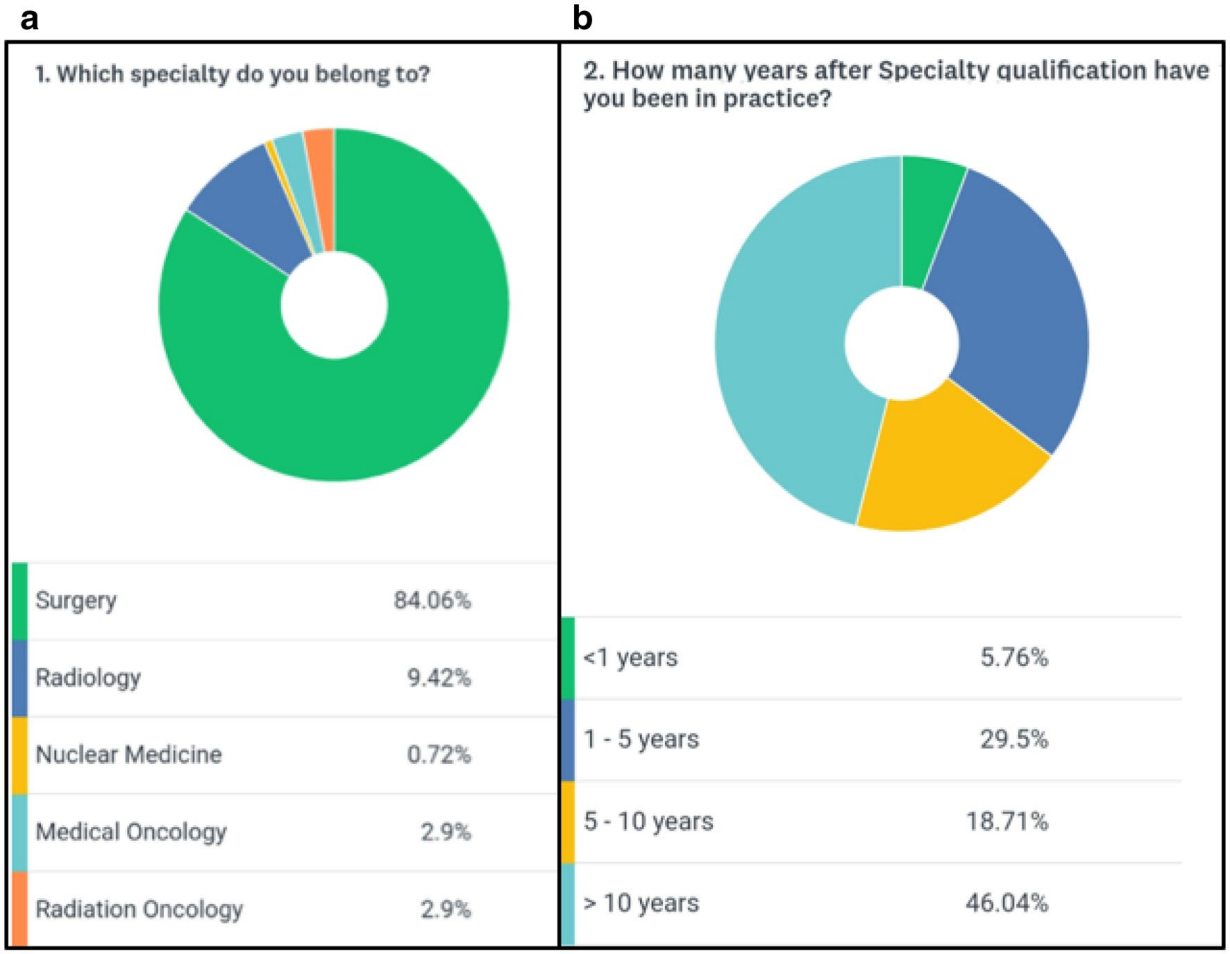
**a** Question no. 1, **b** Question no. 2

**Fig. 2:**
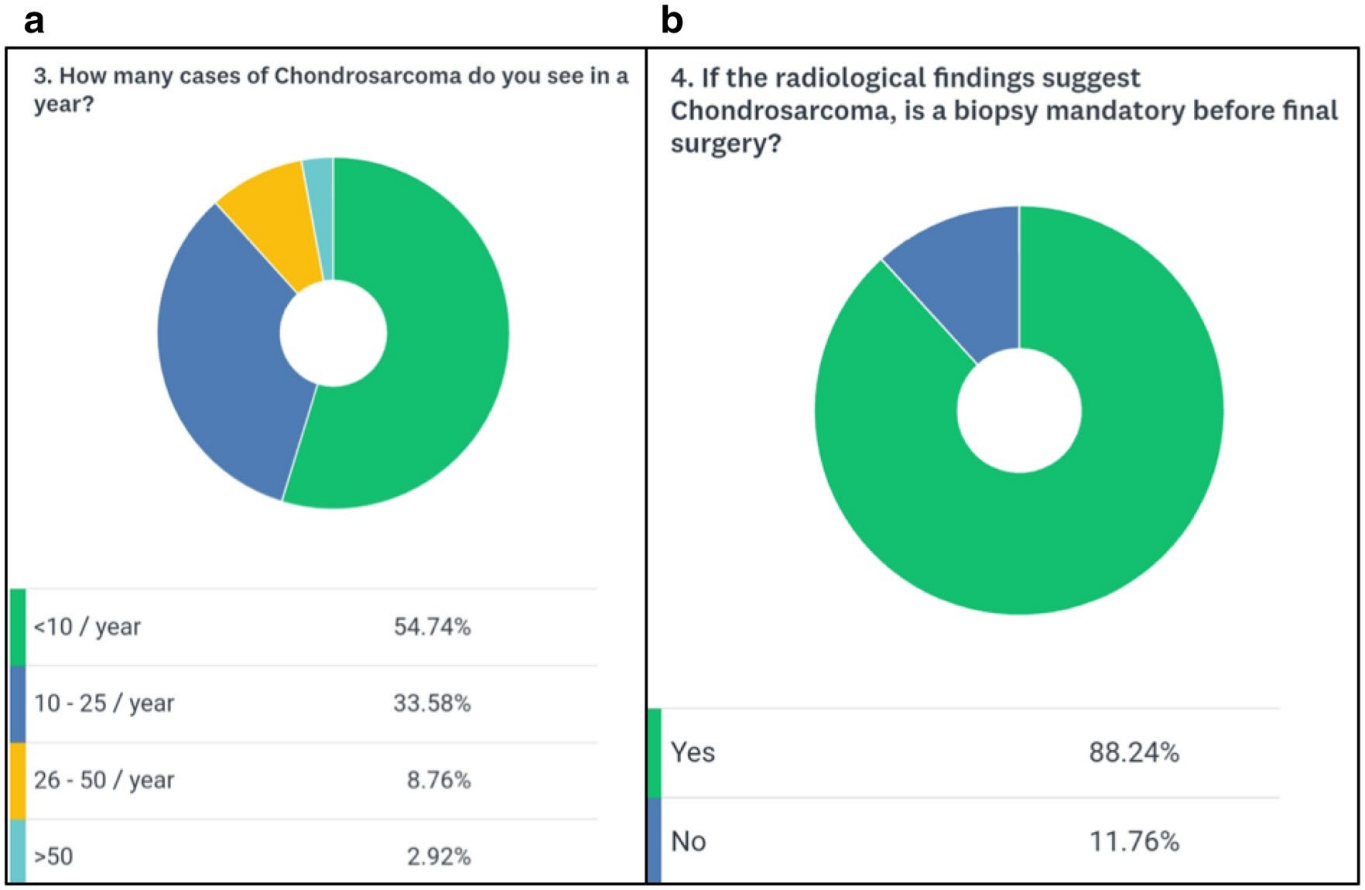
**a** Question no. 3, **b** Question no. 4

**Fig. 3:**
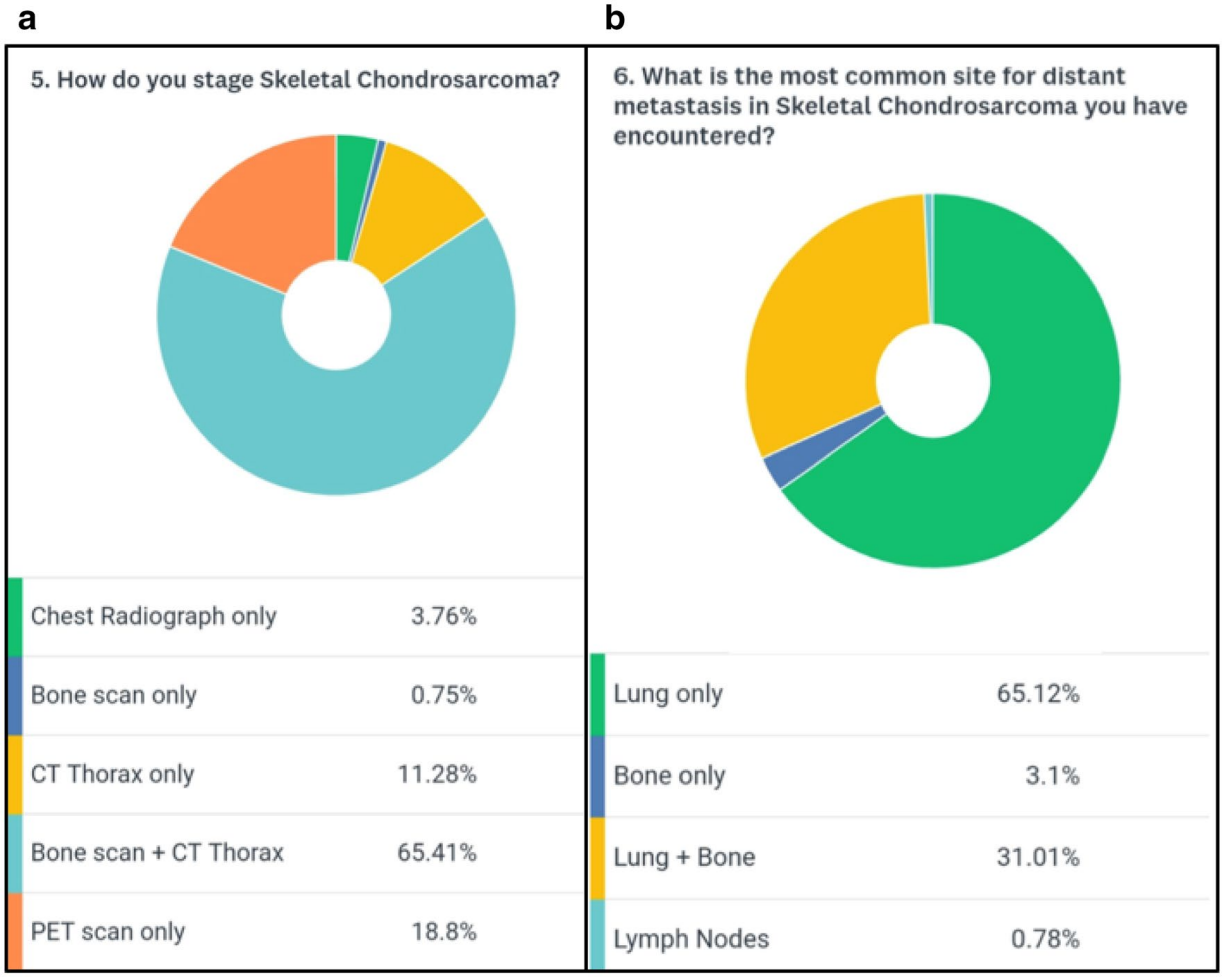
**a** Question no. 5, **b** Question no. 6

**Fig. 4:**
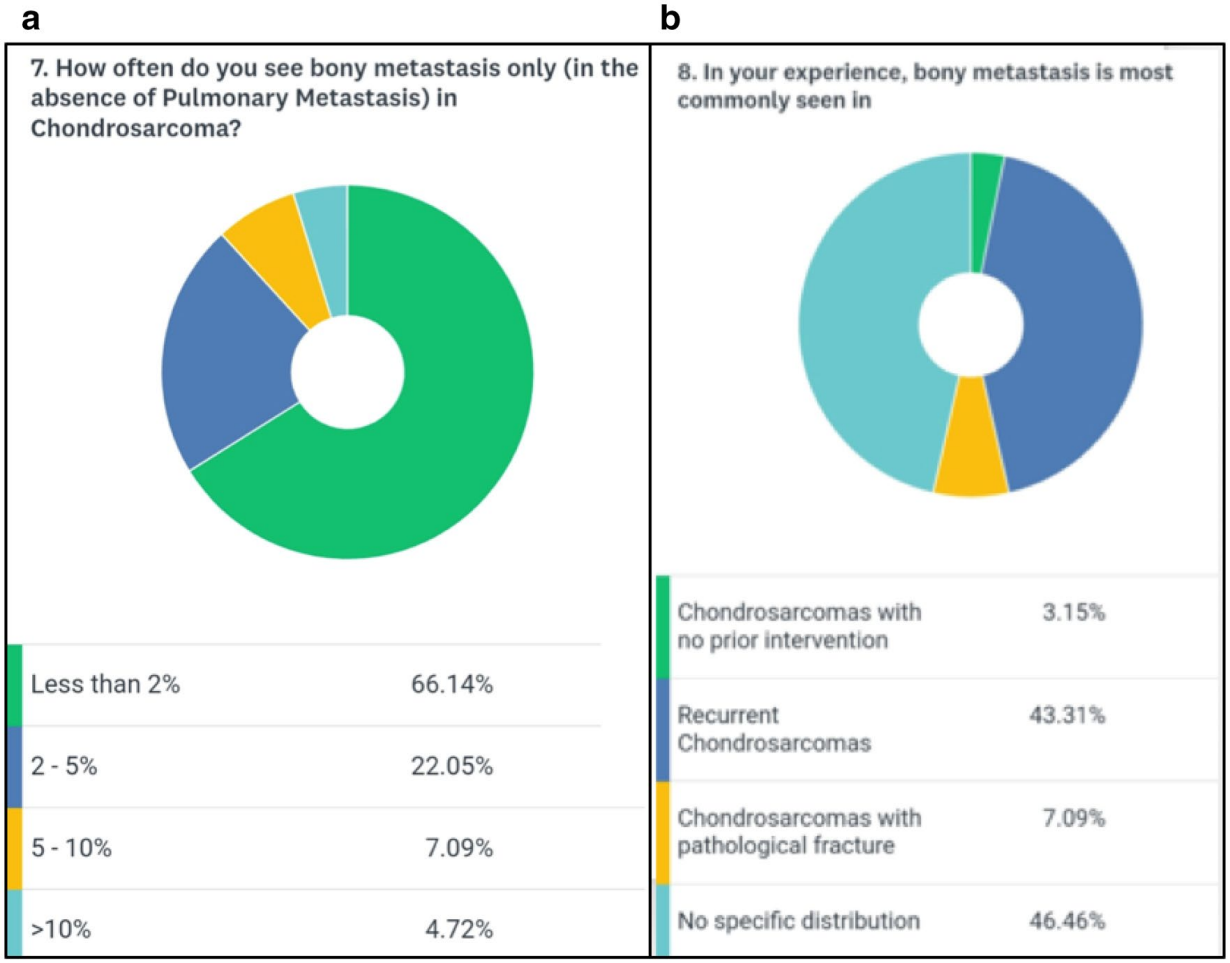
**a** Question no. 7, **b** Question no. 8

**Fig. 5:**
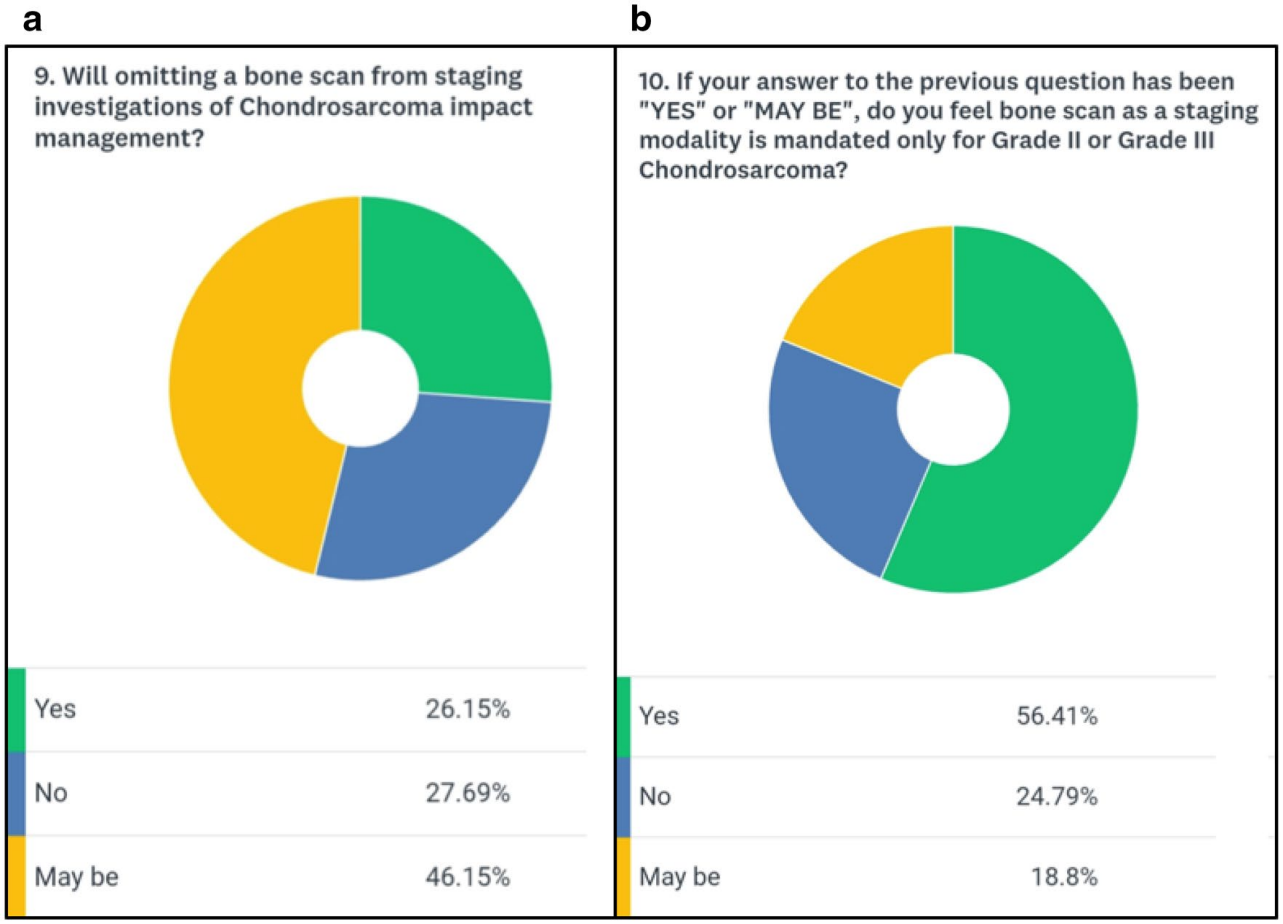
**a** Question no .9, **b** Question no. 10

A total of 139 medical professionals took part in the survey. The majority of participants (84.6%) were surgeons, highlighting the surgical role in the management of chondrosarcomas (Fig. 1a). The combined clinical experience of the respondent cohort was 1144 years (mean 8.2 years) with 65% of participants having oncological experience of more than 5 years (Fig. 1b). The majority of participants (88%) evaluated less than 25 cases of skeletal chondrosarcoma per year (Fig. 2a). Surprisingly 12% of participants said that they would not perform a biopsy before the final surgical intervention (Fig. 2b). 65% of the participants were of the opinion that pulmonary metastasis was the most common site for metastasis in chondrosarcoma whereas 31% were in favor of combined pulmonary and skeletal sites (Fig. 3b). About 88% of participants opined that the percentage of isolated bony metastasis (in the absence of pulmonary metastasis) was less than 5% and 66% were of the opinion that this rate was even lesser than 2% (Fig. 4a). In spite of recognizing the fact that skeletal metastasis was rare in chondrosarcoma, 84% of participants still perform an investigation (bone scan/PET scan) for skeletal survey (Fig. 3a). Only 15% of participants did not feel the need for skeletal staging and performed only chest imaging to detect metastasis (Fig. 3a). About 43% of participants were of the opinion that bony metastasis was more commonly seen in recurrent chondrosarcoma, 3% opined that it is more with prior surgical intervention, 7% believed that a pathological fracture predisposes to skeletal metastasis while about 47% believed that there were no specific reasons for it (Fig. 4b). Only 26% of participants were of the opinion that omitting bone scan as part of the staging workup will impact management while 46% were not sure of its impact (Fig. 5a). Among those answered yes or maybe, 56% of participants were in favor of bone scan as a staging modality being mandatory only for high-grade (Grades 2 and 3) chondrosarcoma while 44% were either not sure or did not feel the need for skeletal staging in high-grade chondrosarcoma (Fig. 5b).

## Discussion

Chondrosarcomas behave differently from other bone sarcomas in being slow growing and having a peak incidence beyond the 4th decade of life [2]. There is less likelihood of metastasis and with adequate surgery, they generally have a good prognosis. Due to their extracellular matrix, poor vascularity and low percentage of dividing cells, chondrosarcomas are relatively chemo- and radioresistant tumors [7].

Staging plays an important role in the management of musculoskeletal tumors by contributing to prognostic evaluation and thus influencing decisions on surgical management and the use of adjuvant therapies [8]. In spite of being different in terms of biologic behavior, response to adjuvant modalities and prognosis, chondrosarcomas are staged in a manner similar to other bone sarcomas. Both, NCCN and ESMO [4, 5], guidelines recommend staging chondrosarcoma with a mix of bone scan, NCCT thorax or FDG-PET. Apart from the financial burden, the radiation dose associated with a FDG-PET–CT is higher than other imaging modalities and is associated with a substantial risk of cancer [9, 10].

Our survey, targeting a very selective population (only those involved in the management of extremity sarcomas), was devised with an aim to determine how chondrosarcomas are staged amongst the oncology fraternity. With a collective clinical experience of 1144 years (mean years of clinical experience 8.2 years), the results of the survey are hard to ignore. In our analysis, 88% of medical personnel encounter less than 25 cases per year which reinforces the fact that chondrosarcomas are a rare entity accounting for only 10–20% of all malignant bone tumors [11].

Conventionally, once there is radiological suspicion of a sarcoma, it is mandatory to confirm it with a histopathological diagnosis [7]. We were surprised to observe that 12% of clinicians did not recommend a biopsy in chondrosarcomas and were satisfied with a radiological diagnosis before proceeding with treatment. Though biopsy is recommended in a suspicious case of chondrosarcoma [12, 13], it may not be reliable for grading [14, 15] and can result in inadequate surgery [16]. This highlights the critical role of clinical history and radiological imaging in chondrosarcoma. Cortical expansion, thickening or destruction and a soft tissue mass usually indicate a high-grade, aggressive tumor [17]. International guidelines do not advocate any surgical intervention without performing a biopsy in a suspicious case of chondrosarcoma [4]. We believe that a core needle biopsy from the most representative area identified after adequate imaging (MRI ±) is the ideal way. Inappropriate evaluation and deviations from standard protocol can lead to compromised outcomes [18] and hence should not be advocated, especially as a part of guidelines which are the recommendations followed by a majority of practicing surgeons.

The majority (65%) mentioned that lung was the most common site for distant metastasis and 95% concurred to screen the lungs with a CT scan [6, 19]. Surprisingly, only 16% were willing to ignore performing a skeletal survey to look for skeletal metastasis in spite of the published literature that did not support the routine use of whole body bone scintigraphy in the initial staging of chondrosarcoma [6, 19].

Skeletal metastasis in the absence of pulmonary metastasis is extremely rare with a through literature search revealing six cases wherein only one case was at index presentation [6, 20–24]. Though there was concurrence on this by two-thirds of the respondents, there was no clear consensus if it had any specific distribution pattern. 43% were of the opinion that skeletal metastases were more common in recurrent cases and 75% believed that bone scan is mandatory while dealing with Grade 2 or 3 chondrosarcoma. Though two-thirds believed that the lung alone was the most common site for metastases, 72% of them were of the opinion that omitting a bone scan might impact the management of chondrosarcoma. The results clearly show an absence of consensus amongst the beliefs of the participants and their application while managing a case of chondrosarcoma. This could be a reflection of the limited literature available [6, 23, 25, 26] and lack of clarity in guidelines formulated [4, 5].

## Conclusion

The present survey results demonstrate the disparity between published literature, the beliefs of practicing musculoskeletal oncologists and current modalities used in staging of chondrosarcoma. The absence of distinctive guidelines for chondrosarcoma staging [4, 5] suggests the need for further studies to determine the ideal staging algorithm for chondrosarcoma: one which balances the cost and side effects without compromising on adequate disease pick-up for optimum oncological care.
